# Sustaining capacity building and evidence-based NCD intervention implementation: Perspectives from the GRIT consortium

**DOI:** 10.3389/frhs.2022.891522

**Published:** 2022-07-22

**Authors:** Ashlin Rakhra, Shivani Mishra, Angela Aifah, Calvin Colvin, Joyce Gyamfi, Gbenga Ogedegbe, Juliet Iwelunmor

**Affiliations:** ^1^New York University Grossman School of Medicine, New York, NY, United States; ^2^New York University School of Global Public Health, New York, NY, United States; ^3^Department of Behavioral Sciences and Health Education, College for Public Health & Social Justice, Saint Louis University, St. Louis, MO, United States

**Keywords:** implementation science, sustainability, non–communicable diseases, LMICs (low and middle income countries), capacity building, evidence-based interventions

## Abstract

**Background:**

Implementation science has been primarily focused on adoption of evidence-based interventions, and less so on sustainability, creating a gap in the field. The Global Research on Implementation and Translation Science (GRIT) Consortium is funded by the National Heart Lung and Blood Institute (NHBLI) to support the planning, implementation, and sustainability of Late-Stage Phase 4 Translational Research (T4TR) and capacity building for NCD prevention and control in eight low-and middle-income countries (LMICs). This paper highlights perspectives, including barriers, facilitators, opportunities, and motivators for sustaining capacity building and evidence-based hypertension interventions within LMICs.

**Methods:**

Guided by the Capacity, Opportunity, Motivation, Behavior (COM-B) Model, this study surveyed GRIT consortium members on the barriers, facilitators, key motivators, and opportunities for sustaining capacity building and evidence-based hypertension interventions in LMICs. Thematic analysis was used to identify themes and patterns across responses.

**Results:**

Twenty-five consortium members across all eight sites and from various research levels responded to the survey. Overarching themes identifying facilitators, key motivators and opportunities for sustainability included: (1) access to structured and continuous training and mentorship; (2) project integration with existing systems (i.e., political systems and health systems); (3) adaption to the local context of studies (i.e., accounting for policies, resources, and utilizing stakeholder engagement); and (4) development of interventions with decision makers and implementers. Barriers to sustainability included local policies and lack of infrastructure, unreliable access to hypertension medications, and lack of sufficient staff, time, and funding.

**Conclusion:**

Sustainability is an important implementation outcome to address in public health interventions, particularly as it pertains to the success of these initiatives. This study provides perspectives on the sustainability of NCD interventions with a focus on mitigating their NCD burden in LMICs. Addressing multilevel factors that influence the sustainability of capacity building and interventions will have notable implications for other global NCD efforts going forward. Current and future studies, as well as consortium networks, should account for sustainability barriers outlined as it will strengthen program implementation, and long-term outcomes.

## Background

The burden of non–communicable diseases (NCDs) continues to rise globally with a disproportionate impact in low and middle-income countries (LMIC) (1). Deaths due to NCDs in LMICs are expected to increase from 30.8 million in 2015 to 41.8 million by 2030 (2). To address this growing disease burden, continued evidence-based interventions (EBI) addressing NCDs and capacity building for NCD investigators in LMICs is needed. Moreover, comprehensive sustainability efforts addressing barriers and facilitators to NCD EBIs and capacity building uptake are crucial to maximize the impact of these efforts to ensure long-term health outcomes are maintained (3, 4).

While program sustainability is not a new concept, the field of implementation science has focused more so on understanding factors and strategies that influence the adoption and implementation of EBIs and less so on the factors and strategies impacting sustainability (5). While studies have discussed multi-level factors influencing sustainability that relate to context (i.e., outer context, policies, legislation, funding and inner context, culture, structure), innovation or the intervention itself (i.e., fit, effectiveness), process (i.e., fidelity, monitoring, evaluation), political support, funding partnerships research on sustainability factors still needs to be more widely adopted (6, 7).

The Global Research on Implementation and Translational Science (GRIT) Consortium was convened in 2018 by the National Heart Lung and Blood Institute (NHLBI) to support the planning, implementation, and sustainability of Late-Stage Phase 4 Translational Research (T4TR) and capacity building initiatives for NCD prevention and control in LMICs. The overarching goal of the GRIT Consortium is to define and establish a strategy that connects consortium members to capacity-building initiatives that will enhance the sustainable uptake of evidence-based interventions for NCD prevention and control in LMICs (2, 8). The network comprises investigators funded by the Hypertension Outcomes for T4 Research in LMICs (HyTREC) and the Translation Research Capacity Building Initiative in LMICs (TREIN) programs. The consortium consists of research teams from eight countries, five of which (Guatemala, Ghana, Kenya, India, and Vietnam) test implementation strategies to deliver evidence-based interventions within these countries for the prevention, treatment, and control of hypertension (HyTREC sites) and three of which (Malawi, Nepal, and Rwanda) provide capacity building in NCD and D&I research needed to close the gap between research and practice (TREIN sites). Additional details of each site in the consortium are published elsewhere and can be found in Table 1 (9–16).

**Table 1:** Summary of GRIT Consortium Sites.

**Site & Project Title**	**Brief Summary of project**
*HyTREC Sites*		
Ghana (10) *Uptake of Task-Strengthening Strategy for Hypertension Control within Community Health Planning Services in Ghana: A Mixed Method Study*	The goal of this study is to evaluate, in a hybrid clinical effectiveness- implementation cluster design, the effect of practice facilitation (PF) on the uptake of an evidence-based Task Strengthening Strategy for Hypertension control (TASSH), among 700 adults who present to 70 Community- Based Health Planning Services (CHPs) zones with uncontrolled hypertension.
Guatemala (14) *Implementing a Multicomponent Intervention to Improve Hypertension Control in Central America. A Cluster Randomized Trial in Guatemala*	A cluster randomized clinical trial to test the co-primary objectives: The effect of a multilevel and multicomponent intervention program on blood pressure (BP) control among Guatemalan hypertensive patients over an 18-month period The acceptability, adoption, feasibility, fidelity, reach, and sustainability of implementing the intervention in patients, providers, and health districts.
India (15) *Integrated Tracking, Referral, and Electronic Decision Support, and Care Coordination (I-TREC)*	The overall goal of this 5-year project is to adapt, implement, and evaluate an IT- enabled platform for integrated tracking, referral, electronic decision support, and care coordination (I-TREC) to treat hypertension and diabetes in rural communities that rely on public health care system using mixed methods approach (Quasi-experimental design).
Kenya (13) *Strengthening Referral Networks for Management of Hypertension Across the Health System (STRENGTHS) in western Kenya: a study protocol of a cluster randomized trial*	A cluster randomized control trial evaluating the effectiveness and cost- effectiveness of a combined health information technology (HIT) and peer support intervention on referral completion, BP improvement, and CVD risk reduction in Kenya.
Vietnam (12) *Conquering Hypertension in Vietnam: Solutions at Grassroots level*	A cluster randomized controlled trial to evaluate the implementation and effectiveness of two multi-faceted community and clinic-based strategies for the control of hypertension among adults residing in the rural Red River Delta region of Vietnam with uncontrolled hypertension.
*TREIN Sites*		
Malawi (16) *NCD BRITE- Building Research Capacity, Implementation and Translation Expertise for non-communicable diseases*	The proposed program will build long-term, sustainable heart, lung, blood and sleeping diseases and disorders (HLBS) focused late-stage translation phase 4 research (T4TR) capacity in Malawi and will utilize this capacity together with research infrastructure and diseases burden needs assessments, to design a Malawi specific HLBS T4TR research plan. The trans-disciplinary consortium is purposefully designed to build capacity within the University of Malawi-College of Medicine (COM), the only public medical school in the country, and the Malawi Ministry of Health (MoH), to ensure sustainability.
Nepal (9) *Translational Research Capacity Building Initiative to Address Cardiovascular Diseases in Nepal*	Dhulikhel Hospital Kathmandu University Hospital, Nepal, will lead work to create a multi-sectoral, multidisciplinary collaborative team to develop Translational research capacity Building initiatives to prevent and manage CVD in Nepal. By the end of the project, we will have developed a critical mass of human Resources in Nepal, collaborating with national and International partners, to conduct Translational Research in CVD. We will have defined clearly identified prioritized needs and a well- defined Translational Research plan to address one or more major CVD Risk Factors and outcomes.
Rwanda (11) *Developing T4 translational research capacity for control of hypertension in Rwanda*	This project will create a collaborative team of academics, clinicians, community healthcare providers, and public health experts to engage in T4TR by building the competencies required to enhance uptake of proven interventions for control of hypertension in Rwanda.

The GRIT Consortium's contribution to hypertension and other NCD knowledge and services is unique due to the collaborative stakeholder and implementer perspectives of multiple LMICs. The GRIT Consortium sites have identified common determinants and adoptable strategies for NCD interventions and capacity building in LMICs (2, 8). The consortium not only addresses the knowledge gap between program implementation and sustainability, but also lays a groundwork for discussing other potential gaps in dissemination and implementation (D&I) practice in LMICs.

As the TREIN and HyTREC projects are in their final phases, the consortium has been focused on sustaining both the capacity building and intervention implementation efforts. For this study, we adapted the Michie and colleagues Behavior Change Wheel framework and the COM-B Model as the model uses three factors- capabilities, opportunities, motivations for identifying changes to ensure behavior change interventions are effective (17, 18). The COM-B Model has been used in other studies addressing the implementation of hypertension interventions in LMICs, as well as in other contexts to develop effective interventions (19). The goal of this study was to examine the capabilities, opportunities, and motivators for sustaining hypertension and other NCD intervention implementation and capacity building in LMICs (17, 18). This study describes barriers, facilitators, motivators and opportunities identified by GRIT Consortium researchers to enhance future NCD sustainability efforts.

## Methods

### Study design and procedure

This was a qualitative open-ended descriptive online survey conducted across the GRIT Consortium in March and April of 2021. This study used purposive sampling to recruit researchers across study roles and from all eight GRIT Consortium sites (Ghana, Guatemala, India, Kenya, Malawi, Nepal, Rwanda, and Vietnam). The survey remained open until saturation was reached.

### Conceptual framework

**Figure 1:**
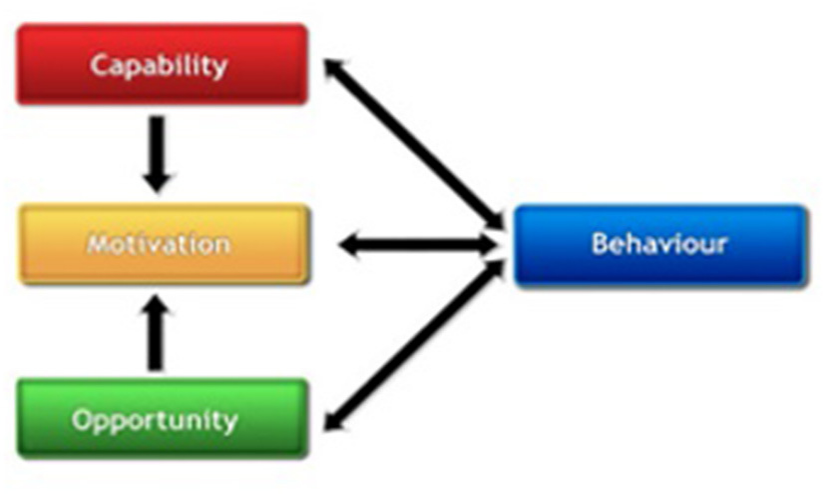
The COM-B model.

Implementing and sustaining behavior changes (i.e., NCD control and capacity building) may occur as a result of an interaction between three components: capability, opportunity, and motivation (6). As such, the survey tool was guided by the Capabilities, Opportunities, Motivations, and Behavior (COM-B) Model (17, 18). Capability is defined as one's psychological capacity (i.e., knowledge) and physical capacity (i.e., skills) to engage in a behavior; Opportunity represents external factors that affect one's capacity to perform (i.e., physical environment, social influences and cultural norms); and Motivation represents internal factors that allow one to employ capability and opportunity to perform a behavior (i.e., wants, needs, beliefs, intentions) (see Figure 1) (17, 18).

### Survey development

Guided by the COM-B model, a qualitative open-ended descriptive online survey was developed and administered to the GRIT consortium project sites. The initial survey was piloted among a sub-group of GRIT members and was subsequently revised and refined based on feedback to ensure clarity of wording and usability. In addition to demographic questions regarding the researchers' role on their study, research team members were asked about their experience as program implementers across the eight countries in the consortium. The survey assessed the three main domains of the COM-B through open-ended questions: (1) what would you say makes it easy or difficult to implement and sustain capacity building and/ or evidence-based HTN intervention implementation? (capability); (2) what would you say motivates researchers/ and or other key stakeholders to implement and sustain capacity building and/ or evidence-based hypertension intervention implementation? (motivation); and (3) what opportunities exist to continuously support researchers/ community members to implement and sustain capacity building and/ or evidence-based hypertension intervention implementation? (opportunities).

### Data analysis

The survey tool was administered in English which was the common language among participants. The data relevant to each construct of the COM-B Model was documented by two authors using a data extraction sheet. The information was summarized and reported descriptively using content analysis to the COM-B Model. Discrepancies between the two authors were resolved by open discussions and consultation sessions among the research team. The consolidated criteria for reporting qualitative studies (COREQ) was followed (20).

## Results

### Participant characteristics

Twenty-five consortium members completed the questionnaire. Table 2 outlines the country site and study team roles of respondents within the consortium. 56 percent of respondents were principles investigators (PIs) or co-investigators (Co-Is), 24% were coordinators, and the remaining respondents included statistician(s), data manager(s), researcher(s), program manager(s), and an international liaison. All eight sites in the consortium were represented in the responses. Table 2 outlines additional respondent demographics.

**Table 2:** Respondent demographics (*n* = 25).

**Country**	
Ghana	12% (3)
Guatemala	12% (3)
India	12% (3)
Kenya	8% (2)
Malawi	12% (3)
Nepal	8% (2)
Rwanda	24% (6)
Vietnam	12% (3)
**Study Team Role**	
PI/ Co-PI	20% (5)
Investigator/ Co-Investigator	36% (9)
Statistician	4% (1)
Data Manager	4% (1)
Coordinator (research, project, implementation)	24% (6)
Researcher	4% (1)
Program Manager	4% (1)
Lead International Liaison	4% (1)

### Barriers and facilitators

Table 3 outlines the respondent-identified barriers and facilitators to sustaining capacity building and/ or evidence-based hypertension or NCD intervention implementation. Barriers identified by respondents included: (1) lack of hypertension medications (17%); (2) lack of time (during implementation and post-intervention) (19%); (3) lack of funding (11%); (4) lack of staff (17%); (5) low education or understanding of intervention/ disease among population/ patient and provider (17%); (6) context (local policies, lack of infrastructure, context specific social and cultural beliefs.) (11%); (7) lack of hypertension diagnosis (3%); (8) lack of epidemiology data (3%); and (9) insufficient or lack of internet access at work (3%). Facilitators included: (1) training opportunities (22%); (2) mentorship and leadership support (11%); (3) community/stakeholder engagement (17%); (4) working in multi-disciplinary teams (8%); (5) local context (adoption to and capacity of local systems) (17%); (6) political support (6%); (7) motivation of staff (3%); (8) quarterly workshops to review challenges in EBI hypertension interventions (3%); and 9) acceptance of hypertension (less stigma, not infectious, modifiable risk factor) (8%).

**Table 3:** Barriers and facilitators to implementing and sustaining capacity building and/or evidence-based NCD interventions.

**Barriers (*n* = 36)**	
Lack of HTN medication	17% (6)
Lack of time	19% (7)
Lack of funding	11% (4)
Lack of staff	17% (6)
Low education (population/ patient and provider)	17% (6)
Context (policies, infrastructure, etc.)	11% (4)
Late diagnosis of HTN	3% (1)
Lack of epidemiology data	3% (1)
Insufficient or lack of internet access at work	3% (1)
**Facilitators (n=36)**	
Training	22% (8)
Mentorship/ Leadership Support	11% (4)
Community/ Stakeholder Engagement	17% (6)
Multi- disciplinary teams	8% (3)
Local Context (adoption to and capacity of systems)	17% (6)
Political Support	6% (2)
Motivation of staff	3% (1)
Quarterly workshops to review challenges in evidence-based hypertension interventions	3% (1)
Hypertension (less stigma, not infectious, modifiable risk factors)	8% (3)

### Key motivators

**Figure 2:**
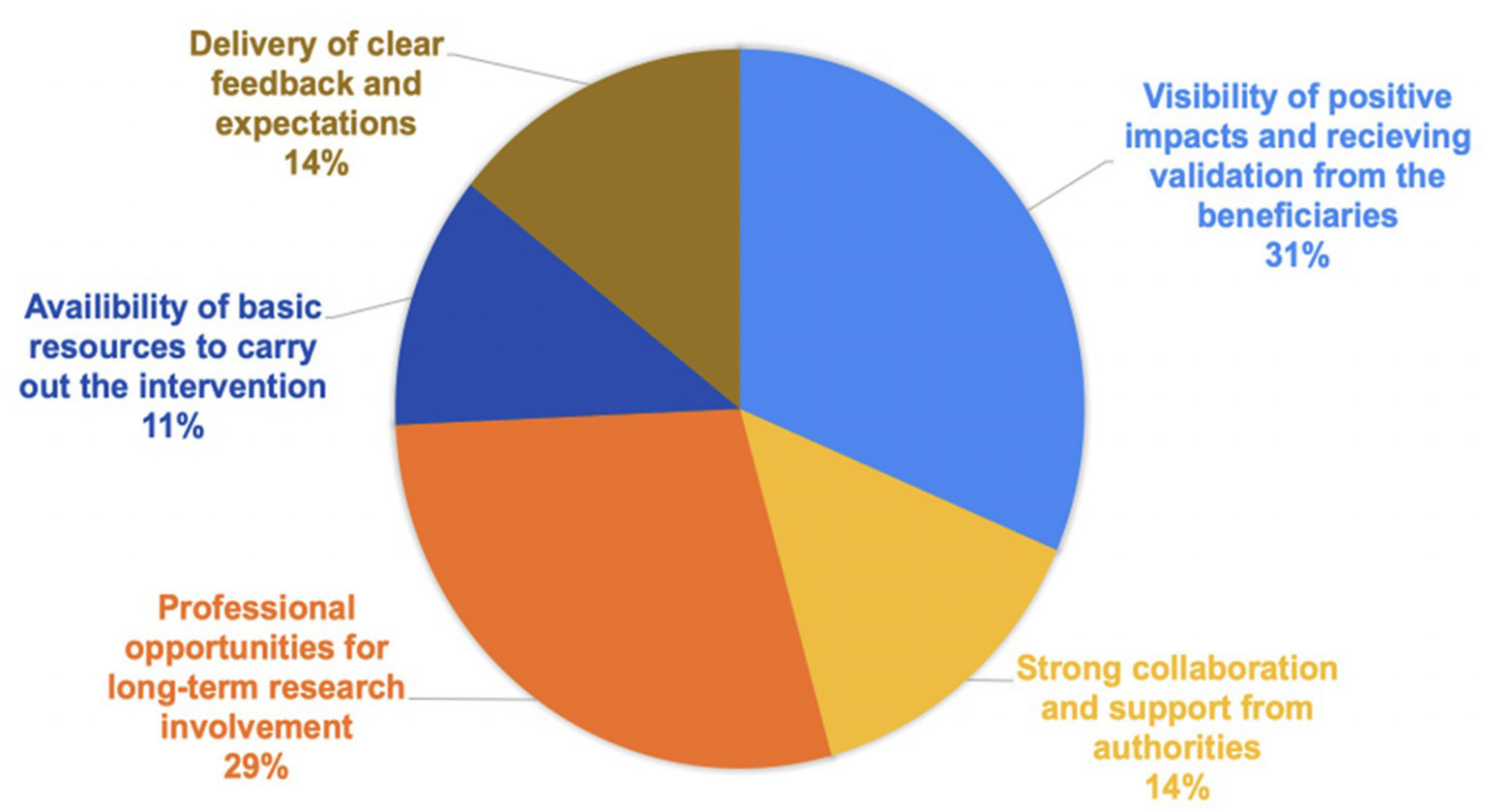
Motivators to Sustaining Capacity-Building and/or Evidence-Based NCD Intervention Implementation.

Figure 2 highlights the most-common motivators for sustaining capacity building and/ or evidence- based NCD intervention implementation. 31 percent of the respondents suggested visibility of positive impacts and receiving validation from beneficiaries; and 29% of respondents suggested professional opportunities for long term research involvement (i.e., salary support, pathways to promotion, sharing new opportunities, etc.) were key motivators driving sustainable interventions. Additional motivators included delivery of clear feedback and expectations (11%), strong collaborations from authorities (i.e., local government officials, local researchers, stakeholders) (14%), and availability of basic resources to carry out the intervention (i.e., minimal funding, administrative and research software, logistics) (11%).

### Opportunities

**Figure 3:**
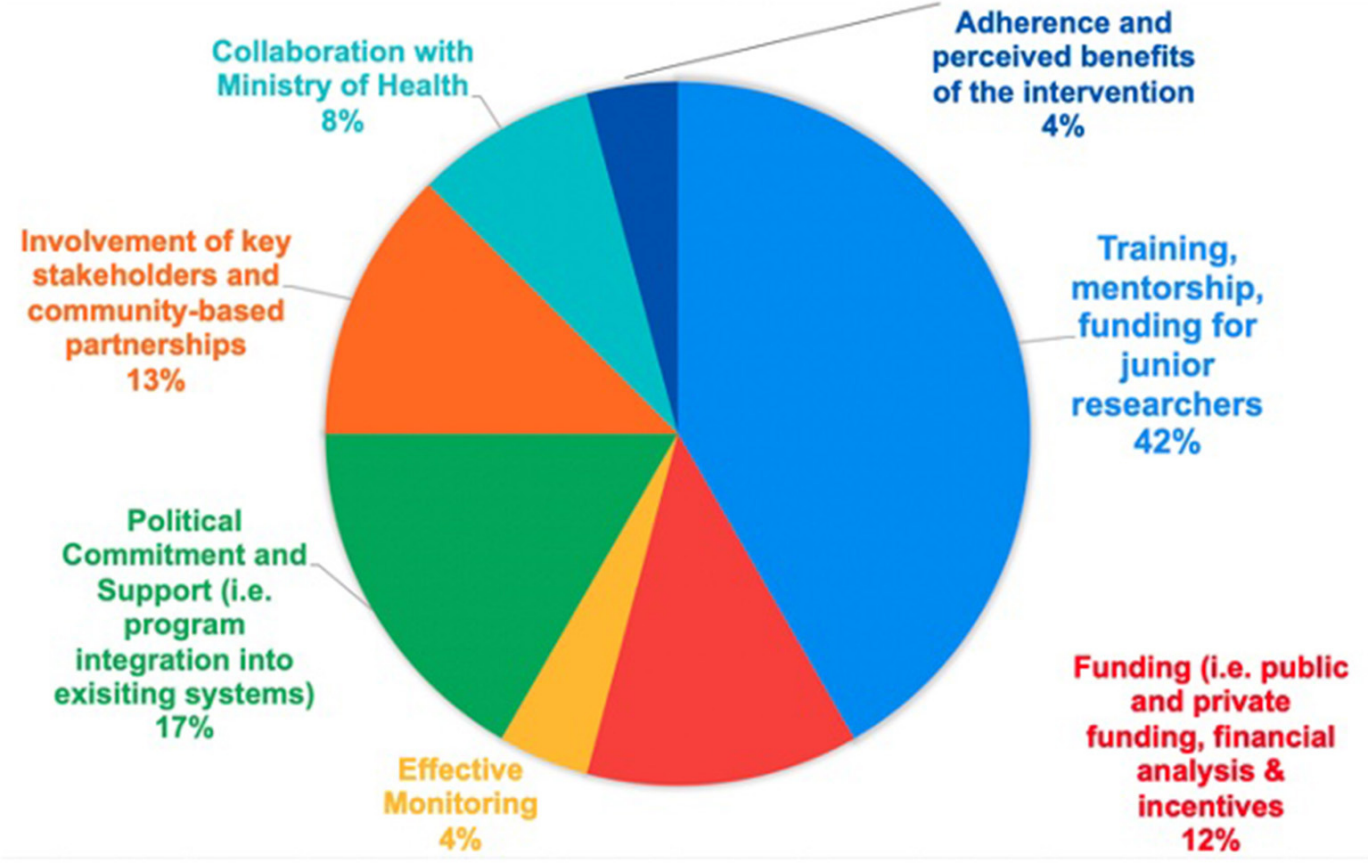
Opportunities to Continuously Support Researchers/Community Members to Implement and Sustain Capacity-Building and/or Evidence-Based NCD Interventions.

Opportunities to support researchers and community members implementing and sustaining capacity building and evidence-based NCD intervention implementation are outlined in Figure 3. 42% of responses included training, mentorship, and funding for junior researchers, followed by 21% of involvement of key stakeholders (i.e., community-based partnerships, Ministry of Health), followed by 17% identifying political commitment and support. Funding (i.e., public and private funding, financial analysis & incentives) (12%), effective monitoring (4%), and adherences and perceived benefits of the intervention were also identified as areas of opportunity (4%).

## Discussion

This study examined the capabilities, opportunities and motivations for sustaining capacity building and evidence-based NCD intervention implementation across eight LMICs. Our study is in accordance with other research findings that discuss multi-level factors that impact sustainability such as political support, funding stability, partnerships, and program evaluation and adaptation (3, 6, 7). Overall, these findings highlight the need for commitment from the various stakeholders including research funding agencies, national and local governments, national and global philanthropy and multilateral organizations to make progress in LMIC research capacity for NCDs (3, 21).

While there was high diversity in respondents, with over 50% being PIs or Co-Is, the need for training, mentorship and funding for junior or early researchers was a prominent theme among all respondents. Training and mentorship are proven strategies that lead to scientific success for junior researchers (22). The lack of support for early-stage investigators in LMICs interested in the global NCD field has resulted in numerous barriers (22), many of which were reported in the findings of this study, including lack of sufficient staff, lack of knowledge among providers and researchers on the research process as well as addressing the interplay between local contextual setting factors. Studies have consistently shown that LMIC investigators are best positioned to address health challenges given their understanding of context, such as the cultural and political climate and health system readiness, in their home countries (5). While there is growing effort for access to training and mentorship to be a long-term goal of projects and institutions (16, 22, 23), including the uptake from TREIN sites within the GRIT Consortium (22, 24, 25), increased access to mentorship still need to be adopted more widely to continue building local research capacity.

An additional finding of this study highlighted in the Integrated Sustainability Framework is the visibility of positive impacts and receiving validation from beneficiaries (5). This included seeing improved health and well–being, building capacity of healthcare workers, and appreciation as motivators for sustaining the work they are doing (22). Visibility of the program impact can be addressed through comprehensive evaluation with a focus on process measures (26). An additional way in which implementers can see positive impacts and receive validation of their efforts is to openly connect with the communities they work with (22). Nearly a quarter of responses identified the involvement of key stakeholders such as community-based partnerships, and Ministry of Health (MOH) as an opportunity to continuously support researchers and community members. Stakeholder involvement in intervention implementation not only encourages community support and creates a program that is more likely to be sustained due to changed community social norms and increased usage (2, 27), but would allow researchers to engage with stakeholders on the program impact.

In addition to stakeholder engagement, partnering with policy makers and financing institutions in the planning and implementation of NCD research and capacity building is crucial for securing funding or other resources needed for the continuation of sustainability efforts post intervention (6). Involving policy makers and funding agencies when developing implementation programs and research to consider sustainability allows for more appropriate planning and allocation of funds potentially resulting in a much better understanding of why and how some interventions and programs last and others do not (4). Lastly, engaging with policy makers and funding agencies could address limited national funding and financial barriers that reduce access to hypertension medications in this study as well as in others (28, 29).

### Implications and recommendations

Sustaining EBIs remains challenging, especially in LMICs where resources may be scarce. Based in eight countries across three continents, the current study adds renewed perspectives on how sustainability can be planned for, and considered in implementation research, which has received limited scientific attention– particularly in LMIC contexts. Findings from this study may serve as a springboard to identify specifically where implementation gaps exist and where targeted strategies are necessary. Findings also points to the need for equitable participation and stakeholder engagement with implementation practitioners and research funders to exchange knowledge on what influences sustainability throughout the life cycle of an EBI and to understand the values of the organization/health system that supports the sustainability of EBIs. Future research consortia may consider supplements or non-competitive funding opportunities to advance both knowledge and action related to the sustainability of evidence-based NCD interventions in LMICs.

### Strengthens and limitations

This study has a number of strengths including the use of data and implementer/ researcher perspectives from eight LMICs, making the findings more generalizable. Second, the study was guided by the COM-B Model. Limitation of this study include the small sample size of survey responses. Additionally, the results were self-reported by respondents thus needing to be validated in a study of long-term project sustainability. Lastly, the structure of the survey grouped both capacity building and intervention implementation in the same questions. While these could have been surveyed as separate concepts, the structure and sustainability of the GRIT Consortium addresses both capacity building and intervention implementation as integrated approaches.

## Conclusion

This study describes the perspectives from key implementers of capacity building and NCD intervention implementation efforts across eight low-and-middle income countries. This study addresses a gap in literature by examining the sustainability of evidence-based NCD implementation. Addressing multilevel factors that influence the sustainability of capacity building and interventions will have notable implications for other global NCD efforts going forward. Current and future studies, as well as consortium networks, should account for sustainability barriers and facilitators outlined as it will strengthen program implementation and long-term outcomes.

## Data availability statement

The raw data supporting the conclusions of this article will be made available by the authors, without undue reservation.

## Author contributions

JI developed the survey using the COM-B Model. AR coordinated survey distribution and data collection. AR and SM conducted the content analysis and drafted the manuscript. SM, AA, and CC all provided feedback on manuscript sections. All authors reviewed and approved the final manuscript. All authors contributed to the article and approved the submitted version.

## Funding

This study was supported by a grant from the National Heart, Lung and Blood Institute (NHLBI), (1-U01 HL 138638).

## Conflict of interest

The authors declare that the research was conducted in the absence of any commercial or financial relationships that could be construed as a potential conflict of interest.

## Publisher's note

All claims expressed in this article are solely those of the authors and do not necessarily represent those of their affiliated organizations, or those of the publisher, the editors and the reviewers. Any product that may be evaluated in this article, or claim that may be made by its manufacturer, is not guaranteed or endorsed by the publisher.

## Abbreviations

COM-B: Capabilities, Opportunities, Motivations, and Behavior Model
Co-Is: Co-Investigators
D&I: Dissemination and Implementation
EBI: Evidence-Based Interventions
GRIT: Global Research on Implementation and Translation Science
HyTREC: Hypertension Outcomes for T4 Research within Lower Middle-Income Countries
LMICs: Low and middle-income countries
NCDs: Non–communicable diseases
NHLBI: National Heart Lung and Blood Institute
PIs: Principle Investigators
T4TR: Late-Stage Phase 4 Translational Research
TREIN: Translation Research Capacity Building Initiative in Low Income Countries.
